# Standardized phenology monitoring methods to track plant and animal activity for science and resource management applications

**DOI:** 10.1007/s00484-014-0789-5

**Published:** 2014-01-25

**Authors:** Ellen G. Denny, Katharine L. Gerst, Abraham J. Miller-Rushing, Geraldine L. Tierney, Theresa M. Crimmins, Carolyn A. F. Enquist, Patricia Guertin, Alyssa H. Rosemartin, Mark D. Schwartz, Kathryn A. Thomas, Jake F. Weltzin

**Affiliations:** 1 National Coordinating Office, USA National Phenology Network, 1955 East Sixth Street, Tucson, AZ 85721 USA; 2 School of Natural Resources and the Environment, University of Arizona, Tucson, AZ 85721 USA; 3 Schoodic Education and Research Center and Acadia National Park, National Park Service, Winter Harbor, ME 04693 USA; 4 SUNY College of Environmental Science and Forestry, Syracuse, NY 13210 USA; 5 The Wildlife Society, Bethesda, MD 20816 USA; 6 Department of Geography, University of Wisconsin-Milwaukee, Milwaukee, WI 53201 USA; 7 U.S. Geological Survey, Southwest Biological Science Center, Tucson, AZ USA; 8 U.S. Geological Survey, Tucson, AZ USA

**Keywords:** Animal, Climate change, Methods, Monitoring, Phenology, Plant, Protocol

## Abstract

Phenology offers critical insights into the responses of species to climate change; shifts in species’ phenologies can result in disruptions to the ecosystem processes and services upon which human livelihood depends. To better detect such shifts, scientists need long-term phenological records covering many taxa and across a broad geographic distribution. To date, phenological observation efforts across the USA have been geographically limited and have used different methods, making comparisons across sites and species difficult. To facilitate coordinated cross-site, cross-species, and geographically extensive phenological monitoring across the nation, the USA National Phenology Network has developed in situ monitoring protocols standardized across taxonomic groups and ecosystem types for terrestrial, freshwater, and marine plant and animal taxa. The protocols include elements that allow enhanced detection and description of phenological responses, including assessment of phenological “status”, or the ability to track presence–absence of a particular phenophase, as well as standards for documenting the degree to which phenological activity is expressed in terms of intensity or abundance. Data collected by this method can be integrated with historical phenology data sets, enabling the development of databases for spatial and temporal assessment of changes in status and trends of disparate organisms. To build a common, spatially, and temporally extensive multi-taxa phenological data set available for a variety of research and science applications, we encourage scientists, resources managers, and others conducting ecological monitoring or research to consider utilization of these standardized protocols for tracking the seasonal activity of plants and animals.

**Electronic supplementary material:**

The online version of this article (doi:10.1007/s00484-014-0789-5) contains supplementary material, which is available to authorized users.

## Introduction

Phenology is the study of the seasonally recurrent activity of plants and animals, such as the timing of plant flowering or bird migration, and is central to understanding ecological interactions in the natural and modified systems upon which human society depends. Contemporary climate change has resulted in widespread and ongoing shifts in phenology across many taxa and within varied geographic regions (Parmesan 2006; Cleland et al. 2007; Thackeray et al. 2010). Such shifts in species’ phenologies can affect ecosystem processes and functioning. For instance, changes in plant and animal phenology have been linked to shifts in timing of wildfires (Westerling et al. 2006), disease (Grulke 2011), carbon cycling (Keeling et al. 1996; Richardson et al. 2009; Hufkens et al. 2012), species interactions (van Asch and Visser 2007; Burkle et al. 2013), and the distribution and abundance of species (Both et al. 2006; Willis et al. 2008; Chuine 2010). In addition, changes in phenology can result in alterations to agricultural practices (Hu et al. 2005; Wolfe et al. 2005; Schwartz et al. 2006), allergy seasons (Van Vliet et al. 2002; Ziska et al. 2011), and the timing of cultural activities, such as blossom festivals (Aono and Kazui 2008; Chung et al. 2011) and public visitation to national parks (Buckley and Foushee 2012).

Numerous studies examining long-term phenological records from the past 50 to 100 years have demonstrated clear changes in the timing of phenological events in taxa including birds, plants, butterflies, and mammals (Inouye et al. 2000; Peñuelas et al. 2002; Gordo and Sanz 2009; Thackeray et al. 2010; Bartomeus et al. 2011; Ovaskainen et al. 2013). These studies have primarily documented advances in spring plant and animal activity, though changes in summer and autumn events have also been documented (Vitasse et al. 2009; Ibanez et al. 2010; Crimmins et al. 2011; Fridley 2012). Together, these analyses underscore the value of phenology data as an indicator of plant, animal, and ecosystem response to climate change (IPCC 2007).

The majority of studies documenting phenological change have been geographically and taxonomically limited. Further, prior studies have used a wide range of methods, including a variety of categories and definitions for various phenological stages, different criteria for determining the presence of such stages, different sampling methods and frequencies, and different units of observation (e.g., plots, individual plants or animals, etc.), making comparative analyses across studies and species challenging (Root et al. 2003; Parmesan 2007; Thackeray et al. 2010; Tooke and Battey 2010; Menzel et al. 2011; Cook et al. 2012; Diez et al. 2012; Wolkovich et al. 2012). A geographically extensive set of observations, collected using standardized protocols on a regular interval, would offer a much stronger data resource for documenting species’ responses to changing climate conditions.

The community of scientists, resource managers, and educators engaged in the USA National Phenology Network (USA-NPN), which was established in 2007, recognized an opportunity for better coordination in phenology data collection (Schwartz et al. 2012b). To this end, members of this group developed a standardized and conceptually integrated method for observing phenology of both plants and animals that can be implemented across polar, temperate, tropical, and water-limited ecosystems. As outlined below, this method includes several elements to enhance the detection and description of phenological responses beyond what is possible with some other methods commonly used in temperate regions in the past. By encouraging those initiating new observation efforts to follow these protocols—and existing observation efforts to develop crosswalks or adopt aspects of the protocols where appropriate—we hope to build a large, spatially and temporally extensive, freely available, phenological data set based on a common sampling method.

Here, we present the general monitoring approach and specific phenophases developed by scientists working as part of the USA-NPN. These protocols are designed for in situ observations of plant and animal phenology across terrestrial, freshwater, and marine ecological systems. We define the term *phenophase* as an observable stage or phase in the annual life cycle of a plant or animal that can be characterized by a start and an end point. Phenophases typically have a duration of a few days or weeks. Examples of phenophases include the period over which newly emerging leaves are visible or the period during which frogs are calling.

## Attributes and advantages of monitoring methods

These standardized protocols are designed to quantify the onset, duration, and intensity of phenological stages of plants and animals to understand how life cycles track environmental variation. The protocols can be tailored to any sampling density or frequency depending on available resources and the science or management question under investigation, and enable integrated monitoring of both plants and animals to address questions related to both populations and communities. The phenophase categories associated with particular life forms and functional types are summarized in Tables 1, 2, 3, 4, and corresponding phenophase definitions are outlined in detail in Online Resource 1. The monitoring method is characterized by several elements that allow for enhanced detection and description of phenological responses through time including (1) repeated assessment of phenophase “status” to provide explicit information on presence as well as absence of a phenophase, (2) intensity or abundance of phenophases, (3) independent tracking of different and potentially overlapping phenophases, and (4) monitoring of multiple individuals within a population.

**Table 1:** Summary of USA-NPN phenophases for plants

	Phenophase title	Cactus	Conifer (general)	Conifer (pine)	Conifer (deciduous)	Forb (general)	Forb (evergreen)	Grass/sedge/rush	Tree/shrub (deciduous)	Tree/shrub (drought deciduous)	Tree/shrub (semievergreen)	Tree/shrub (broadleaf evergreen)
Vegetative phenophases	Initial growth					X		X				
	Breaking leaf buds								X		X	X
	Young leaves						X			X	X	X
	Leaves					X		X	X	X	X	
	Increasing leaf size								X		X	
	Colored leaves								X	X	X	
	Falling leaves								X		X	
	Breaking needle buds		X		X							
	Emerging needles			X								
	Young needles		X	X								
	Needles				X							
	Colored needles				X							
	Falling needles				X							
Reproductive phenophases	Flowers or flower buds	X				X	X	X	X	X	X	X
	Open flowers	X				X	X	X	X	X	X	X
	Pollen release	X	X	X	X	X	X	X	X	X	X	X
	Pollen cones		X	X	X							
	Open pollen cones		X	X	X							
Fruit/seed phenophases	Fruits	X				X	X	X	X	X	X	X
	Ripe fruits	X				X	X	X	X	X	X	X
	Recent fruit or seed drop	X				X	X	X	X	X	X	X
	Unripe seed cones		X	X	X							
	Ripe seed cones		X	X	X							
	Recent cone or seed drop		X	X	X							

Column headings represent plant functional groups, and row headings are phenophases to be observed. An “X” indicates this phenophase should be observed for species in that functional group. For phenophase definitions and more detailed information about the USA-NPN protocols, see Online Resource 1

**Table 2:** Summary of USA-NPN phenophases for insects

Phenophase title	Mayfly	Dragonfly/damselfly	Grasshopper	Stonefly	Tiger beetle	Butterfly	Moth	Bee
Active adults	X	X	X	X	X	X	X	X
Adults feeding		X	X		X			
Flower visitation						X		X
Migrating adults		X				X		
Mating	X	X	X	X	X	X	X	X
Egg laying		X						
Active subadults	X							
Active caterpillars						X	X	
Caterpillars in tent							X	
Caterpillars feeding						X	X	
Dead caterpillars						X	X	
Active nymphs			X					
Nymphs feeding			X					
Dead nymphs			X					
Dead adults	X	X	X	X	X	X	X	X
Individuals at a feeding station						X	X	
Individuals at a light					X		X	
Individuals in a net	X	X	X	X	X	X	X	

Column headings represent insect guilds, and row headings are phenophases to be observed. An “X” indicates this phenophase should be observed for species in that guild. Note that phenophases for advanced insect observers are not included here, and protocols have yet to be developed for several important insect taxa. For phenophase definitions and more detailed information about the USA-NPN protocols, see Online Resource 1

**Table 3:** Summary of USA-NPN phenophases for fish, amphibians, and reptiles

Phenophase title	Fish (saltwater)	Fish (anadromous)	Fish (freshwater)	Eel	Salamander	Toad/frog	Alligator	Turtle	Lizard/snake
Individuals on land							X	X	X
Adults on land					X	X			
Individuals in water							X	X	X
Adults in water					X	X			
Adults in freshwater		X	X	X					
Adults in saltwater	X	X		X					
Feeding							X	X	X
Adults feeding	X	X	X	X	X	X			
Adults migrating upstream		X							
Adults migrating downstream		X	X	X					
Juveniles in saltwater	X								
Juveniles moving upstream				X					
Vocalizing						X			
Adults vocalizing							X		
Mating					X	X			
Nesting								X	
Fresh eggs					X	X			
Young individuals							X	X	X
Dead individuals							X	X	X
Dead adults					X	X			
Dead or dying adults	X	X	X	X					
Individuals on a hook	X	X	X	X					
Individuals in a net	X	X	X	X					

Column headings represent animal guilds, and row headings are phenophases to be observed. An “X” indicates this phenophase should be observed for species in that guild. Note that phenophases for advanced animal observers are not included here. For phenophase definitions and more detailed information about the USA-NPN protocols, see Online Resource 1

**Table 4:** Summary of USA-NPN phenophases for birds and mammals

Phenophase title	Bird (general)	Shorebird	Hummingbird	Songbird	Mammal (general)	Pinniped	Squirrel/chipmunk	Deer/sheep
Active individuals	X	X	X	X	X		X	X
Individuals on land						X		
Individuals in water						X		
Feeding	X	X	X	X	X	X	X	X
Fruit/seed consumption	X			X			X	
Insect consumption	X		X	X				
Flower visitation			X	X				
Nut gathering	X						X	
Calls or song	X	X	X	X				
Singing males			X	X				
Males vocalizing								X
Male combat						X		X
Mating	X	X	X	X	X	X		X
Nest building	X		X	X				
Young individuals					X	X	X	X
Summer coat					X			
Winter coat					X			
Dead individuals	X	X	X	X	X	X	X	X
Individuals at a feeding station	X		X	X				

Column headings represent animal guilds, and row headings are phenophases to be observed. An “X” indicates this phenophase should be observed for species in that guild. Note that phenophases for advanced animal observers are not included here. For phenophase definitions and more detailed information about the USA-NPN protocols, see Online Resource 1

**Fig. 1:**
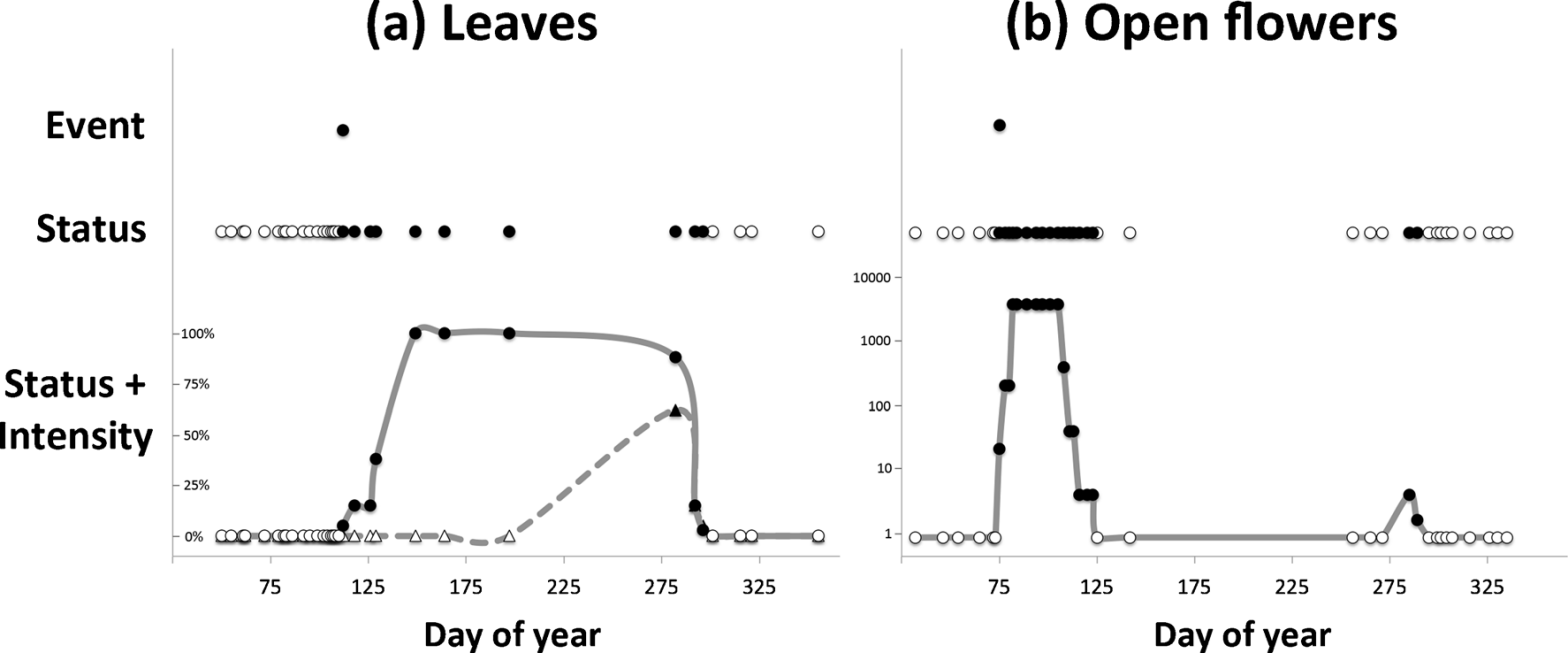
Visual comparison of data collected by monitoring phenological events, phenophase status, and phenophase status plus intensity. Event monitoring captures onset of a given phenophase, whereas status monitoring captures onset and duration. Status monitoring with intensity (or abundance) captures onset, duration, and magnitude of a phenophase. Examples are derived from 2012 data submitted in *Nature’s Notebook* for (**a**) sugar maple (*Acer saccharum*) leafing for one individual plant in Maine, and (**b**) forsythia flowering (*Forsythia sp.*) for one individual plant in Massachusetts. Each *point* represents one observation; *black points* indicate presence of the phenophase while *white points* indicate absence. (**a**) illustrates the date on which the first leaf appears (event), the period during which leaves are present (status), and the period and rate at which the canopy fills from 0 to 100 % capacity and then, empties back to 0 with leaf fall (status + intensity, *circles* and *solid line*) using estimates of canopy fullness. Also illustrated is the period and rate at which the canopy fills and empties of autumn colored leaves (status + intensity, *triangles* and *dashed line*). (**b**) illustrates the date on which the first open flower appears (event), the periods during which open flowers are present on the plant (status), and an estimate of the number of open flowers on the plant over the periods in which they are present (status + intensity). In both examples, the event point is calculated as the first date of the year where the phenophase was reported as present. Note that in (**b**) there are two distinct periods of flowering, the second of which would not have been captured using event monitoring alone

The first key element that defines the monitoring method is the periodic assessment of the “status” of the phenophase for an organism, rather than simply recording the date of an “event” (Fig. 1). Historically, many individuals and phenological monitoring programs have recorded the timing of phenological *events*—that is, precisely defined points in the annual life cycles of plants or animals (e.g., Sparks and Carey 1995; Bradley et al. 1999; Fitter and Fitter 2002; Miller-Rushing and Primack 2008). Examples of phenological *events* include first leaf and first flower of plant individuals or species, or first arrival and first departure of migratory animal species. Event data have been instrumental in documenting changes in spring leaf and flower onset in many studies, as well as changes in migration timing and species interactions (e.g., Bradley et al. 1999; Inouye 2008; McKinney et al. 2012). Data collected via a status monitoring approach can offer even more information and further insight into species’ phenology than can be gleaned from event monitoring. For instance, event-based monitoring generally misses repeat events (e.g., a second flush of leaves after a killing frost or a second round of flowering within a season (Crimmins et al. 2013), Fig. 1b). As such, event-based monitoring is impractical in tropical or subtropical systems where the beginning (or end) of a season or a phenophase is often difficult to define. For this reason, monitoring methods in tropical regions have long employed continuous assessment of phenophases (Morellato et al. 2010). Moreover, event-based monitoring (e.g., first frog call of the season or first hummingbird at a feeder) does not necessarily reflect the population-level behaviors of interest to resource managers (Miller-Rushing et al. 2008a, b).

**Fig. 2:**
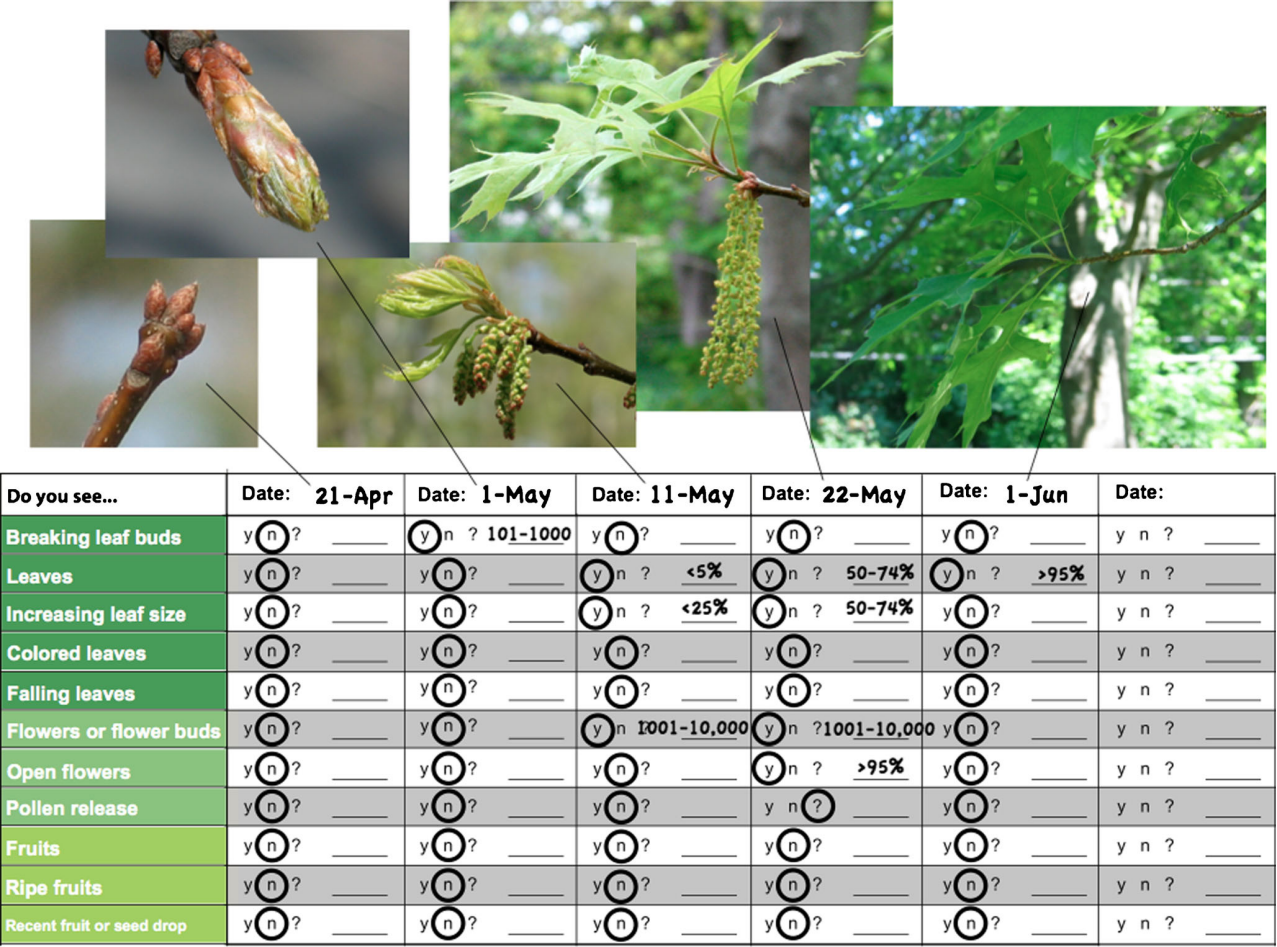
An illustration of how an observer would make and record repeated observations for a single individual plant (in this case a black oak tree, *Quercus velutina*) over a period of time. *Circles* around the “y’s” (yes) and “n’s” (no) indicate the presence or absence of the phenophases (far left column) on the tree for each date. When a phenophase is present, an estimate of intensity is included (see Online Resource 1 for intensity measures). In this example, the phenological event of “first leaf” (Meier 2001) would have occurred between May 1st and May 11th, the dates of the last reported “no” and the first reported “yes” for the “Leaves” phenophase. Although there are approximately 10 days between each observation in this example, more frequent observation will be desired in many cases

Instead of recording the date of phenological events directly, *status* monitoring involves evaluating phenophase *status* (e.g., the presence or absence of leaves, flowers, or fruits for plants, and mating, feeding, or movement for animals) during a series of repeated observations over the course of a season (e.g., Frankie et al. 1974; Inouye and McGuire 1991; Borchert 1994; Sparks et al. 2005; Morellato et al. 2010; Crimmins et al. 2011) (Fig. 1). Observations are expressed as the question, “Do you see [phenophase]?” to which the observer answers “yes”, “no”, or “uncertain” for the presence of each phenophase (Fig. 2). Depending on frequency of observation, this approach provides explicit information on presence, absence, and duration of phenophases, as well as any within-season gaps in the presence of a phenophase resulting from periodic or repeated activity (e.g., flushes of leaves or flowers, or pulses of migratory animals) (Fig. 1b). This approach also enables conceptual and actual integration of the observation of sessile (e.g., plants) and mobile (e.g., birds) organisms at the same location, making it possible, for example, to explicitly record whether pollinators are present while flowers are open or whether leaves are present at the time caterpillars hatch. In sum, this integrated multi-taxa approach creates an integrated framework for tracking phenology of both plants and animals at the level of either populations or communities.

Phenophase *status* monitoring also allows determination of phenological *event* dates from the resulting data, depending on the application or information needs of the user. For example, the date of the first report of presence after a report of absence for open flowers can be interpreted to reflect the event date of “first flower”, though the user can define custom criteria for defining events. Alternatively, event-based data can be mapped onto status-based data; for example, historical event-based observations of lilacs were integrated into the status-based database of the USA-NPN, extending the spatial and temporal range of the USA-NPN database (Schwartz et al. 2012a).

In situations where observations are impossible to make everyday (e.g., remote locations), status-based monitoring provides a mechanism to quantify the uncertainty in the event date by capturing the frequency of observation (e.g., the number of days that passed between the last report of absence and the first report of presence). Status monitoring enables observers to record data each time they make an observation (i.e., “yes,” “no,” or “uncertain”), which can be more active and engaging than event-based monitoring. Finally, with status monitoring, even a single observation of phenophase presence or absence is potentially useful as it can be combined with observations of other observers.

A second element that characterizes the monitoring method is the inclusion of intensity or abundance measures associated with the presence of a particular phenophase (Figs. 1 and 2). In addition to documenting the presence or absence of a phenophase, observers also may record the intensity or abundance of each phenophase (e.g., number of flowers present, percentage of flowers open, number of robins feeding, etc.). For example, rather than simply collecting data on the presence of open flowers on a given plant, these protocols allow an observer to also document the total number of flowers and the proportion of flowers that are open on a given day. These data could be used to identify periods of low flower abundance that may be important for plant reproduction, for pollinators, or for other flower-dependent species (Miller-Rushing and Inouye 2009; Aldridge et al. 2011). For animal species, recording abundance facilitates detection of important population trends, such as declines or increases within and across years, and the timing of pulses of migration or breeding that might be particularly important for resource managers. Coupled with phenophase status monitoring, these intensity and abundance measures allow researchers to better characterize and model phenological patterns in time and space using metrics such as duration, magnitude, mean and skew (e.g., Thomson 1980). They also facilitate assessments of potential interactions among species (Durant et al. 2005; Miller-Rushing et al. 2010).

The third feature that characterizes the monitoring method is the independent tracking of unique phenophases on an individual plant or animal species regardless of whether or not the phenophases are occurring at the same time. For example, an observer documenting the phenology of a deciduous tree would evaluate several leaf phenophases independently of each other during each observation, including the presence and percentage of any leaves (green or colored) and the presence and percentage specifically of colored (non-green) leaves. Thus, an observer may document the presence of both green and colored leaves on one visit, and the presence of colored leaves and absence of green leaves on a subsequent visit (Fig. 1a). This feature allows for greater flexibility in understanding the complex relationships between climate, environmental cues, and phenology as evaluation of separate phenophase responses permits researchers to tease apart interactions among these variables. For instance, researchers can now evaluate the effect of drought on leaf color change and leaf drop independently. Likewise, a researcher can tease apart the effects of temperature on flower production and the opening of flowers. This creates a richer, more complex data set than other methods that might be designed to determine the single dominant phenophase or phenological condition of an individual organism at a given point in time (e.g., Richardson et al. 2006).

A final defining feature of the monitoring method is that the phenology of individual plants is tracked independently, and multiple individuals of the same species can be observed at the same location. This allows researchers to quantify phenological variation within a population as well as across species and geographic regions. A key gap in our understanding of how species will respond to climate change is predicting the extent to which organisms will be able to keep pace with their changing environment (Gienapp et al. 2008; Hoffmann and Sgrò 2011). Characterizing the range of inter- and intra-specific phenological responses to temporal and spatial variation in climate will allow for better understanding of species capacity to respond to shifting abiotic conditions and improved attribution of observed phenological shifts to evolutionary processes vs. adaptive plastic responses. In particular, these data can be used to complement and inform genetic studies and common garden experiments that aim to elucidate mechanisms of adaptation to a changing climate (e.g., Franks et al. 2007).

## Development and implementation of monitoring methods

The standardized protocols presented herein were developed with an input from a large and diverse community of researchers and resource managers with expertise in phenology, ecology, or climate change science, and/or practical experience in the collection and analysis of human-observed field data of select taxonomic groups. Usability feedback from educators and volunteer observers informed subsequent revisions to the protocols. See www.usanpn.org/plant-animal-credits for a list of all contributors to the protocol development. Objectives used to guide the development of the monitoring approach and phenophase definitions include applicability across a wide range of biomes and species, usability for observers with varying levels of skill (professional scientists and resource managers as well as volunteer observers), and utility for a number of anticipated scientific end-uses of the resulting data, such as detection of changes in the start of spring or autumn leaf color, prediction of allergy seasons, validation of remotely sensed land products, and evaluation and prediction of species range shifts or mismatches in the phenology of species interactions. In addition, the protocols were designed to be compatible with other historical phenology sampling methods, including the European BBCH scale developed for tracking phenology in agricultural systems and now used broadly in monitoring natural systems across Europe (Meier 2001; Koch et al. 2007), as well as existing volunteer-based phenology monitoring programs such as eBird and FrogWatch USA (Schwartz et al. 2013).

These standardized protocols can be used by any program monitoring phenology. As a case example, these protocols are employed in the USA-NPN’s phenology observation program, *Nature’s Notebook* (www.nn.usanpn.org), which engages both professionals and volunteers in observing and recording plant and animal activity across the nation (Schwartz et al. 2012a, b). The resulting data, housed in the USA-NPN’s National Phenology Database, are freely available for download, visualization, exploration, and analysis (http://www.usanpn.org/data) (Rosemartin et al. 2013). Because data were collected in *Nature’s Notebook* during development of these standardized protocols, documentation of modifications is provided for data end-users (www.usanpn.org/results/nndocumentation). Several national level organizations and agencies in the USA are using these protocols for phenology observation at pilot sites and/or are in the process of officially adopting them in their standard operating procedures (Tierney et al. 2013; Haggerty et al. 2013) They are also in use by many regional, state, and local partner organizations (e.g., cooperative extension programs, schools, and conservation organizations).

## Conclusion

As the field of phenology expands and its importance in ecology, evolution, and resource management is better defined, the need for high quality standardized observation methods is becoming increasingly clear. The standardized protocols described here provide an approach to enhance detection and description of phenological responses and facilitate greater integration of phenological data collection efforts across the globe. Researchers are using the data generated from these protocols to address a number of science questions on regional to continental scales (Schwartz et al. 2012b; Euskirchen et al. 2013; Jeong et al. 2013; Liang and Schwartz 2013). Integrated with other types of data relevant to plant and animal phenology (e.g., climate data, satellite and ground-based remote sensing products, physiological and demographic measurements, data on human behaviors, and health issues), data generated with these protocols will expand our ability to carry out collaborative and comparative studies, provide new insights into the causes and consequences of changes in phenology on a broad range of spatial and temporal scales, and significantly advance our understanding of ecosystem functioning and the impacts of climate change. In sum, we encourage those working within and across the fields of ecology, animal behavior, resource management, ecosystem science, and climatology to incorporate phenological monitoring into long-term studies using these status-based phenology protocols.

## Acknowledgments

EGD, AJM, GLT, and JFW contributed to the theoretical framework for the monitoring approach and conceptual development of plant and animal protocols. Additionally, KLG, MDS, TMC, PG, and KAT contributed to the conceptual development of plant protocols, and AHR contributed to the conceptual development of animal protocols. KLG drafted the manuscript text. AJM, GLT, TMC, CAFE, AHR, MDS, KAT, and JFW reviewed, and EGD, TMC, and KLG revised the manuscript. Several anonymous reviewers provided valuable comments that significantly improved the manuscript.

Many individuals contributed to the development and review of these phenology protocols over the last 6 years. These individuals are listed online at www.usanpn.org/plant-animal-credits. The USA-NPN gratefully acknowledges the following sponsoring organizations: The US Geological Survey, University of Arizona, University of Wisconsin–Milwaukee, The Wildlife Society, US National Park Service, National Oceanic and Atmospheric Administration, National Aeronautics and Space Administration, National Science Foundation (Research Coordination Network grant, IOS-0639794), Oak Ridge National Laboratory, and US Fish and Wildlife Service. We are also grateful to the Northeastern States Research Cooperative (through funding made available by the USDA Forest Service) and Microsoft Research for funding the lead author during the initial years of protocol development. Data for Fig. 1 were provided by two of the many participants who contribute to *Nature’s Notebook*.

Any use of trade, product, or firm names is for descriptive purposes only and does not imply endorsement by the US Government.

## Electronic supplementary material

Below is the link to the electronic supplementary material.

ESM 1 (PDF 342 kb)
