# Supplementary material for: Standardized phenology monitoring methods to track plant and animal activity for science and resource management applications
Source: Int J Biometeorol. 2014 Jan 25;58(4):591–601. doi: 10.1007/s00484-014-0789-5 (PMC4023011; doi:10.1007/s00484-014-0789-5)
Supplement: Supplementary file 1 — (PDF 342 kb) [file 484_2014_789_MOESM1_ESM.pdf]

USA National Phenology Network

Plant and Animal Phenophase Definitions

Supplement to:

International Journal of Biometeorology

Standardized phenology monitoring methods to track plant and animal activity for science and resource management applications

Ellen G. Denny*, Katharine L. Gerst, Abraham J. Miller-Rushing, Geraldine L. Tierney, Theresa M. Crimmins, Carolyn A.F. Enquist, Patricia Guertin, Alyssa H. Rosemartin, Mark D. Schwartz, Kathryn A. Thomas and Jake F. Weltzin

*USA-NPN National Coordinating Office; University of Arizona
Email: ellen@usanpn.org
Phone: 207-475-2365

Last updated August 2013

Any use of trade, product, or firm names is for descriptive purposes only and does not imply endorsement by the U.S. Government.

Although this report is in the public domain, permission must be secured from the individual copyright owners to reproduce any copyrighted material contained within this report.

TABLE OF CONTENTS

Plants 3
- Introduction 3
- Summary Tables 5
  - Angiosperms and Gymnosperms 5
  - Historic Cloned and Common Lilac 6
- Angiosperm Phenophase Definitions 6
  - Leaf Phenophases 6
  - Flower Phenophases 8
  - Fruit Phenophases 10
- Conifer Phenophase Definitions 11
  - Needle Phenophases 11
  - Pollen Cone Phenophases 12
  - Seed Cone Phenophases 13
- Historic Cloned and Common Lilac Phenophase Definitions 14
  - Leaf Phenophases 14
  - Flower Phenophases 14
- Frequently Asked Questions About Plant Phenophases 15

Animals 21
- Introduction 21
- Summary Tables 22
  - Insects 22
  - Fishes, Amphibians and Reptiles 23
  - Birds and Mammals 24
- Insect Phenophases 25
- Fish Phenophases 28
- Amphibian Phenophases 29
- Reptile Phenophases 31
- Bird Phenophases 32
- Mammal Phenophases 34

PLANTS

INTRODUCTION

Standardized phenophases developed by the USA-NPN for plants are outlined below. Phenophases in use for each plant group are summarized in the tables, and written definitions are included in the following sections. Each phenophase is posed to the observer as the question "Do you see [phenophase]?" to which the observer records "yes", "no" or "uncertain" for each date and time of observation. Several of the phenophases are nested such that if one is occurring the other must also be occurring. For example, if "Open flowers" are visible on a plant, "Flowers or flower buds" are also visible by definition.

Intensity or abundance measures for each phenophase are included below each definition. These represent either an estimate of the number of plant structures (e.g., buds, flowers, fruits) as one of several quantitative classes (<3, 3-10, 11-100, 101-1,000, 1,001-10,000, >10,000), or an estimate of the percentage of plant structures in a phenophase as one of six percentage ranges (<5%, 5-24%, 25-49%, 50-74%, 75-95%, >95%). The quantitative classes were chosen to represent increasing orders of magnitude, and can be used to track exponential increases and decreases in visible structures (e.g., breaking leaf buds) as plants develop during a growing season, as well as to track year-to-year variability in the total number of structures that are produced during a season (e.g., fruit masting). The six percentage ranges were chosen to represent quartiles (i.e. 25%, 50%, 75%, 100%) as well as the tails of a normal distribution (i.e. 0-5% and 95-100%). Reporting in discrete quantitative classes or percentage ranges is favored over reporting continuous variables or finer-scale percentages (e.g., reporting a bin of "101-1000" instead of "652", or a range of "5-25%" instead of "16%") to simplify observation for observers who might be uncomfortable with or unable to provide such precise estimates, and to optimize observation time for all observers (e.g., given the limited resource of observer time, ten plants with a quick, coarse estimate of intensity are more useful than a single plant with a time-consuming, precise estimate of intensity). Note that some phenophases do not include an intensity or abundance measure.

The phenophases and intensity/abundance measures are designed to be applied to an individual plant (or a delineated patch of clonal or small individual plants of the same species growing en masse). Several individuals (or patches) of the same species can be evaluated separately at a single location for replication.

A major challenge to standardization is the fact that different observers can interpret the meanings of definitions differently. We have made every effort to make the definitions as clear and complete as possible in order to minimize variation in interpretation. To this end, observers should interpret definitions literally in all situations and resist trying to second guess alternative meanings for unanticipated situations they believe the creators of the definitions might have implied but did not clearly state.

The phenophases presented here are designed for an observer audience with intermediate botanical knowledge. Additional phenophases for a more advanced audience exist in draft form and are not included in this document. Please see the USA-NPN website (www.usanpn.org) for future updates to these protocols. Also visit the *Nature's Notebook* website

(www.nn.usanpn.org) to find the specific phenophases recommended for each of over 600 plant species, including some clarifying species-specific descriptions added to phenophase definitions, most notably for fruit phenophases.

Summary Tables

Angiosperms and Gymnosperms

	Phenophase title	Cactus	**Conifer** (general)	**Conifer** (pine)	**Conifer** (deciduous)	**Forb** (general)	**Forb** (evergreen)	**Grass/Sedge/Rush**	**Tree/Shrub** (deciduous)	**Tree/Shrub** (drought-deciduous)	**Tree/Shrub** (semi-evergreen)	**Tree/Shrub** (broadleaf evergreen)
**Vegetative phenophases**	Initial growth					X		X				
	Breaking leaf buds								X		X	X
	Young leaves						X			X	X	X
	Leaves					X		X	X	X	X	
	Increasing leaf size								X		X	
	Colored leaves[a]								X	X	X	
	Falling leaves								X		X	
	Breaking needle buds		X		X							
	Emerging needles[b]			X								
	Young needles		X	X								
	Needles				X							
	Colored needles				X							
	Falling needles				X							
**Reproductive phenophases**	Flowers or flower buds[c]	X				X	X	X	X	X	X	X
	Open flowers	X				X	X	X	X	X	X	X
	Pollen release[d]	X	X	X	X	X	X	X	X	X	X	X
	Pollen cones		X	X	X							
	Open pollen cones		X	X	X							
**Fruit/seed phenophases**	Fruits	X				X	X	X	X	X	X	X
	Ripe fruits	X				X	X	X	X	X	X	X
	Recent fruit or seed drop	X				X	X	X	X	X	X	X
	Unripe seed cones		X	X	X							
	Ripe seed cones		X	X	X							
	Recent cone or seed drop		X	X	X							

[a] excluded for species with no noticeable color change leading up to leaf senescence
[b] "Emerging needles" is included for pines instead of "Breaking needle buds" in order to capture the period when needles unfold from their fascicle sheaths after the bud has broken and the candle has elongated
[c] entitled "Flower heads" for grasses and sedges
[d] in angiosperms, only included for allergen species in *Nature's Notebook*

Historic Cloned and Common Lilac

	Phenophase title
**Vegetative phenophases**	Breaking leaf buds (previously “First leaf”)
	All leaf buds broken (previously “Full leaf out”)
**Reproductive phenophases**	Open flowers (previously “First bloom”)
	Full flowering (previously “Full bloom”)
	End of flowering (previously “Last bloom”)

ANGIOSPERM PHENOPHASE DEFINITIONS

Leaf Phenophases

Initial growth

(***Forb***) New growth of the plant is visible after a period of no growth (winter or drought), either from above-ground buds with green tips, or new green or white shoots breaking through the soil surface. Growth is considered "initial" on each bud or shoot until the first leaf has fully unfolded. For seedlings, "initial" growth includes the presence of the one or two small, round or elongated leaves (cotyledons) before the first true leaf has unfolded.

(***Grass/Sedge***) New growth of the plant is visible after a period of no growth (winter or drought), either as new green shoots sprouting from nodes on existing stems, or new green shoots breaking through the soil surface. For each shoot, growth is considered "initial" until the first leaf has unfolded.

(***Rush***) New growth of the plant is visible after a period of no growth (winter or drought) as new green shoots breaking through the soil surface. For each shoot, growth is considered "initial" until the exposed, green portion of the shoot has reached approximately 2 inches (5 cm) in length.

Breaking leaf buds

(***Tree/Shrub***) One or more breaking leaf buds are visible on the plant. A leaf bud is considered "breaking" once a green leaf tip is visible at the end of the bud, but before the first leaf from the bud has unfolded to expose the leaf stalk (petiole) or leaf base.

How many buds are breaking?
Less than 3; 3 to 10; 11 to 100; 101 to 1,000; 1,001 to 10,000; More than 10,000

Young leaves

(***Forb***) One or more young leaves are visible on the plant. A leaf is considered "young" before it has reached full size or turned the darker green color or tougher texture of mature leaves on the plant. Do not include fully dried or dead leaves.

(***Tree/Shrub***) One or more young, unfolded leaves are visible on the plant. A leaf is considered "young" and "unfolded" once its entire length has emerged from the breaking bud so that the leaf stalk (petiole) or leaf base is visible at its point of attachment to the stem, but before the leaf has reached full size or turned the darker green color or tougher texture of mature leaves on the plant. Do not include fully dried or dead leaves.

How many young leaves are present?
Less than 3; 3 to 10; 11 to 100; 101 to 1,000; 1,001 to 10,000; More than 10,000

Leaves

(***Forb***) One or more live, fully unfolded leaves are visible on the plant. For seedlings, consider only true leaves and do not count the one or two small, round or elongated leaves (cotyledons) that are found on the stem almost immediately after the seedling germinates. Do not include fully dried or dead leaves.

(***Grass***) One or more live, green, unfolded leaves are visible on the plant. A leaf is considered "unfolded" once it unrolls slightly from around the stem and begins to fall away at an angle from the stem. Do not include fully dried or dead leaves.

What percentage of the plant is green?
Less than 5%; 5-24%; 25-49%; 50-74%; 75-94%; 95% or more

(***Sedge***) One or more live, green, unfolded leaves are visible on the plant. A leaf is considered "unfolded" once it has grown long enough that the two halves of the leaf blade have begun to spread apart like an open book. Do not include fully dried or dead leaves.

What percentage of the plant is green?
Less than 5%; 5-24%; 25-49%; 50-74%; 75-94%; 95% or more

(***Rush***) One or more live, green, unfolded leaves are visible on the plant. A leaf is considered "unfolded" once the exposed, green portion of the leaf (or shoot) has reached approximately 2 inches (5 cm) in length. Do not include fully dried or dead leaves.

What percentage of the plant is green?
Less than 5%; 5-24%; 25-49%; 50-74%; 75-94%; 95% or more

(***Tree/Shrub***) One or more live, unfolded leaves are visible on the plant. A leaf is considered "unfolded" once its entire length has emerged from the breaking bud so that the leaf stalk (petiole) or leaf base is visible at its point of attachment to the stem. Do not include fully dried or dead leaves.

What percentage of the canopy is full with leaves? Ignore dead branches in your estimate.
Less than 5%; 5-24%; 25-49%; 50-74%; 75-94%; 95% or more

Increasing leaf size

(***Tree/Shrub***) A majority of leaves on the plant have not yet reached their full size and are still growing larger. Do not include new leaves that continue to emerge at the ends of elongating stems throughout the growing season.

What percentage of full size are most leaves?
Less than 25%; 25-49%; 50-74%; 75-94%; 95% or more

Colored leaves

(***Tree/Shrub***) One or more leaves (including any that have recently fallen from the plant) have turned to their late-season colors. Do not include fully dried or dead leaves that remain on the plant.

What percentage of the canopy is full with colored leaves?
Less than 5%; 5-24%; 25-49%; 50-74%; 75-94%; 95% or more

Falling leaves

(***Tree/Shrub***) One or more leaves are falling or have recently fallen from the plant.

Flower Phenophases

Flowers or flower buds

(***Cactus/Forb/Rush/Tree/Shrub***) One or more fresh open or unopened flowers or flower buds are visible on the plant. Include flower buds that are still developing, but do not include wilted or dried flowers.

How many flowers and flower buds are present? For species in which individual flowers are clustered in flower heads, spikes or catkins (inflorescences), simply estimate the number of flower heads, spikes or catkins and not the number of individual flowers.
(*Cactus/Forb/Rush*) Less than 3; 3 to 10; 11 to 100; 101 to 1,000; More than 1,000
(*Tree/Shrub*) Less than 3; 3 to 10; 11 to 100; 101 to 1,000; 1,001 to 10,000; More than 10,000

Flower heads

(***Grass/Sedge***) One or more fresh flower heads (inflorescences) are visible on the plant. Flower heads, which include many small flowers arranged in spikelets, emerge from inside the stem and gradually grow taller. Include flower heads with unopened or open flowers, but do not include heads whose flowers have all wilted or dried.

How many fresh flower heads are present?
Less than 3; 3 to 10; 11 to 100; 101 to 1,000; More than 1,000

Open flowers

(***Cactus/Forb/Rush/Tree/Shrub***) One or more open, fresh flowers are visible on the plant. Flowers are considered "open" when the reproductive parts (male stamens or female pistils) are visible between or within unfolded or open flower parts (petals, floral tubes or sepals). Do not include wilted or dried flowers.

What percentage of all fresh flowers (buds plus unopened plus open) on the plant are open? For species in which individual flowers are clustered in flower heads, spikes or catkins (inflorescences), estimate the percentage of all individual flowers that are open.
Less than 5%; 5-24%; 25-49%; 50-74%; 75-94%; 95% or more

(***Grass/Sedge***) One or more open, fresh flowers are visible on the plant. A flower is considered "open" when reproductive parts (male anthers or female stigmata) can be seen protruding from the spikelet. Do not include flowers with wilted or dried reproductive parts.

What percentage of all fresh flowers (unopened plus open) on the plant are open?
Less than 5%; 5-24%; 25-49%; 50-74%; 75-94%; 95% or more

Pollen release

(***Cactus/Forb/Grass/Sedge/Rush/Tree/Shrub***) One or more flowers on the plant release visible pollen grains when gently shaken or blown into your palm or onto a dark surface.

How much pollen is released?
Little: *Only a few grains are released.*; **Some:** *Many grains are released.*; **Lots:** *A layer of pollen covers your palm, or a cloud of pollen can be seen in the air when the wind blows.*

Fruit Phenophases

Fruits

(***Cactus/Forb/Grass/Sedge/Rush/Tree/Shrub***) One or more fruits are visible on the plant. *Species-specific description included here.*

How many fruits are present?
(*Cactus/Forb/Grass/Sedge/Rush*) Less than 3; 3 to 10; 11 to 100; 101 to 1,000; More than 1,000
(*Tree/Shrub*) Less than 3; 3 to 10; 11 to 100; 101 to 1,000; 1,001 to 10,000; More than 10,000

Ripe fruits

(***Cactus/Forb/Grass/Sedge/Rush/Tree/Shrub***) One or more ripe fruits are visible on the plant. Species-specific description included here.

What percentage of all fruits (unripe plus ripe) on the plant are ripe?
Less than 5%; 5-24%; 25-49%; 50-74%; 75-94%; 95% or more

Recent fruit or seed drop

(***Cactus/Forb/Grass/Sedge/Rush/Tree/Shrub***) One or more mature fruits or seeds have dropped or been removed from the plant since your last visit. Do not include obviously immature fruits that have dropped before ripening, such as in a heavy rain or wind, or empty fruits that had long ago dropped all of their seeds but remained on the plant.

How many mature fruits have dropped seeds or have completely dropped or been removed from the plant since your last visit?
(*Cactus/Forb/Grass/Sedge/Rush*) Less than 3; 3 to 10; 11 to 100; 101 to 1,000; More than 1,000
(*Tree/Shrub*) Less than 3; 3 to 10; 11 to 100; 101 to 1,000; 1,001 to 10,000; More than 10,000

CONIFER PHENOPHASE DEFINITIONS

Needle Phenophases

Breaking needle buds

(***Evergreen Conifer, excluding Pines***) One or more breaking needle buds are visible on the plant. A needle bud is considered "breaking" once a green needle tip is visible at the end of the bud, but before the first needle from the bud has unfolded and spread away at an angle from the developing stem.

How many buds are breaking?

Less than 3; 3 to 10; 11 to 100; 101 to 1,000; 1,001 to 10,000; More than 10,000

(***Deciduous Conifer***) One or more breaking needle buds are visible on the plant. A needle bud is considered "breaking" once a green needle tip is visible at the end of the bud, but before the first needle from the bud has unfolded and spread away at an angle from the developing stem, or from other needles in a bundle.

How many buds are breaking?

Less than 3; 3 to 10; 11 to 100; 101 to 1,000; 1,001 to 10,000; More than 10,000

Emerging needles

(***Pine***) One or more emerging needles or needle bundles (fascicles) are visible on the plant. A needle or needle bundle is considered "emerging" once the green tip is visible along the newly developing stem (candle), but before the needles have begun to unfold and spread away at an angle from others in the bundle.

How many needles or needle bundles are emerging?

Less than 3; 3 to 10; 11 to 100; 101 to 1,000; 1,001 to 10,000; More than 10,000

Young needles

(***Evergreen Conifer, excluding Pines***) One or more young, unfolded needles are visible on the plant. A needle is considered "young" and "unfolded" once it has spread away from the developing stem enough that its point of attachment to the stem is visible, but before it has reached full size or turned the darker green color or tougher texture of mature needles on the plant.

How many young needles are present?

Less than 3; 3 to 10; 11 to 100; 101 to 1,000; 1,001 to 10,000; More than 10,000

(***Pine***) One or more young, unfolded needles are visible on the plant. A needle is considered "young" and "unfolded" once it begins to spread away at an angle from other needles in the bundle (and is no longer pressed flat against them), but before it has reached full size or turned the darker green color or tougher texture of mature needles on the plant.

How many young needles are present?
Less than 3; 3 to 10; 11 to 100; 101 to 1,000; 1,001 to 10,000; More than 10,000

Needles

(***Deciduous Conifer***) One or more live, unfolded needles are visible on the plant. A needle is considered "unfolded" once it begins to spread away at an angle from the developing stem enough that its point of attachment to the stem is visible, or from other needles in a bundle so that it is no longer pressed flat against them. Do not include fully dried or dead needles.

What percentage of the canopy is full with needles? Ignore dead branches in your estimate.
Less than 5%; 5-24%; 25-49%; 50-74%; 75-94%; 95% or more

Colored needles

(***Deciduous Conifer***) One or more needles (including any that have recently fallen from the plant) have turned to their late-season colors. Do not include fully dried or dead needles that remain on the plant.

What percentage of the canopy is full with colored needles?
Less than 5%; 5-24%; 25-49%; 50-74%; 75-94%; 95% or more

Falling needles

(***Deciduous Conifer***) One or more needles are falling or have recently fallen from the plant.

Pollen Cone Phenophases

Pollen cones

(***All Conifers***) One or more fresh, male pollen cones (strobili) are visible on the plant. Cones have overlapping scales that are initially tightly closed, then spread apart to open the cone and release pollen. Include cones that are unopened or open, but do not include wilted or dried cones that have already released all of their pollen.

How many fresh pollen cones are present?
Less than 3; 3 to 10; 11 to 100; 101 to 1,000; 1,001 to 10,000; More than 10,000

Open pollen cones

(***All Conifers***) One or more open, fresh, male pollen cones (strobili) are visible on the plant. Cones are considered "open" when the scales have spread apart to release pollen. Do not include wilted or dried cones that have already released all of their pollen.

What percentage of all fresh pollen cones (unopened plus open) on the plant are open?
Less than 5%; 5-24%; 25-49%; 50-74%; 75-94%; 95% or more

Pollen release

(***All Conifers***) One or more male cones (strobili) on the plant release visible pollen grains when gently shaken or blown into your palm or onto a dark surface.

How much pollen is released?
Little: *Only a few grains are released.*; **Some:** *Many grains are released.*; **Lots:** *A layer of pollen covers your palm, or a cloud of pollen can be seen in the air when the wind blows.*

Seed Cone Phenophases

Unripe seed cones

(***All Conifers***) One or more unripe, female seed cones are visible on the plant. *Species-specific description included here.*

How many seed cones are unripe?
Less than 3; 3 to 10; 11 to 100; 101 to 1,000; 1,001 to 10,000; More than 10,000

Ripe seed cones

(***All Conifers***) One or more ripe, female seed cones are visible on the plant. *Species-specific description included here.*

How many seed cones are ripe?
Less than 3; 3 to 10; 11 to 100; 101 to 1,000; 1,001 to 10,000; More than 10,000

Recent cone or seed drop

(***All Conifers***) One or more seed cones or seeds have dropped or been removed from the plant since your last visit. Do not include empty seed cones that had long ago dropped all of their seeds but remained on the plant.

How many seed cones have dropped seeds or have completely dropped or been removed from the plant since your last visit?
Less than 3; 3 to 10; 11 to 100; 101 to 1,000; 1,001 to 10,000; More than 10,000

Historic Cloned and Common Lilac Phenophase Definitions

Leaf Phenophases

Breaking leaf buds

(***Lilac***) In at least 3 locations on the plant, a breaking leaf bud is visible. A leaf bud is considered "breaking" once the widest part of the newly emerging leaf has grown beyond the ends of its opening winter bud scales, but before it has fully emerged to expose the leaf stalk (petiole) or leaf base. The leaf is distinguished by its prominent midrib and veins. (*This phenophase was previously called "First leaf".*)

All leaf buds broken

(***Lilac***) For the whole plant, the widest part of a new leaf has emerged from virtually all (95-100%) of the actively growing leaf buds. (*This phenophase was previously called "Full leaf out".*)

Flower Phenophases

Open flowers

(***Lilac***) For the whole plant, at least half (50%) of the flower clusters have at least one open fresh flower. The lilac flower cluster is a grouping of many, small individual flowers. (*This phenophase was previously called "First bloom".*)

Full flowering

(***Lilac***) For the whole plant, virtually all (95-100%) of the flower clusters no longer have any unopened flowers, but many of the flowers are still fresh and have not withered. (*This phenophase was previously called "Full bloom".*)

End of flowering

(***Lilac***) For the whole plant, virtually all (95-100%) of the flowers have withered or dried up and the floral display has ended. (*This phenophase was previously called "Last bloom".*)

FREQUENTLY ASKED QUESTIONS ABOUT PLANT PHENOPHASES

1. Can I still report “Breaking leaf/needle buds” (trees and shrubs), “Emerging needles” (pines), or “Initial growth” (forbs and grasses) once I see “Leaves/Needles” or “Young leaves/needles” on the plant?

Yes, you should judge each leaf bud, needle bud, or shoot separately. As long as some buds or shoots on the plant are still breaking or initiating growth and have not yet produced an unfolded leaf or needle, you are seeing “Breaking leaf/needle buds”, “Emerging needles”, or “Initial growth”. For plants that have more than one bud or shoot, in most cases you will still be seeing “Breaking leaf/needle buds”, “Emerging needles”, or “Initial growth” in some buds or shoots for many days after you first begin seeing “Leaves/Needles” or “Young leaves/needles” from other buds or shoots. It is also possible to see multiple episodes of leaf/needle bud break or initial growth within a season. This might occur after a period of frost, drought, or after a plant is defoliated by insects. However, once ALL the active leaf/needle buds or shoots on the plant have at least one unfolded leaf/needle, you should be reporting that you no longer see “Breaking leaf/needle buds”, “Emerging needles”, or “Initial growth”.

2. How do I judge whether or not a leaf or needle is “young”?

Young leaves or needles on an evergreen or drought-deciduous plant can be distinguished from older leaves or needles because they are smaller, a lighter shade of green, and/or thinner than mature leaves or needles. As the young leaf matures and approaches the size, color and thickness of older leaves on the plant, it can be difficult to determine when to no longer consider it “young”. This is particularly true for drought-deciduous species where new leaf growth is often initiated by rain events. If water becomes unavailable after growth is initiated, leaf expansion may stop, resulting in many small leaves on the plant. If this is the case, you will need to stop reporting that you see “Young leaves/needles” when it appears that the leaves or needles have stopped changing in size, color and thickness (or report that you are “Uncertain” of whether or not you see “Young leaves/needles”). With experience, it will become easier to determine when a leaf or needle is still growing and young.

3. Why is there a phenophase for “Emerging needles” for pine species but not for other conifer species?

In most conifers, breaking needle buds reveal new needles unfolding, then lengthening of the stem, and the unfolding of more needles until the stem stops growing for the season. In pines, growth of the stem (or “candle”) occurs first, before the needles appear. Once the stem has grown, the needle bundles begin to emerge and unfold from protective papery sheaths along the newly elongated stem. We have decided that “Breaking needle buds” should be an advanced phenophase for pine species, and thus do not include it in the protocols at this time. Instead, observation of pine species begins with the search for emerging needles along the new stem.

4. When should I report I no longer see "Leaves/Needles"?

You should continue to report seeing "Leaves/Needles" as long as fresh green or colored leaves/needles remain on the plant. Do not include dried, dead leaves or dead, brown needles that remain on the plant, such as occurs with some species throughout the dormant season (e.g., winter or dry season). In some cases, green leaves will remain on the plant in a frozen condition for part or all of the winter. If more than about 5% of the leaves have remained on the plant in this condition, you should continue to report seeing "Leaves" until they fall off or appear wilted.

5. How do I judge what percentage of the plant is green in grasses, sedges and rushes?

For grass, sedge and rush species where new growth is from new stems, the plant will probably be 100% green until it begins to turn brown in the late summer or fall. For species where existing stems can turn brown and then re-green, the percentage may start low at the beginning of the growing season, become higher in the middle of the growing season, and then decline again as the plant turns brown again. In dryland environments where conditions are extreme, it can be particularly difficult to judge what portion of a grass plant is truly dead and what portion has the potential to re-green. If this is the case for your plant, you may want to ignore this intensity question and just report on whether or not you see green leaves.

6. How do I judge what percentage of the canopy is full with leaves or needles?

To be able to do this requires knowing what a full canopy looks like for any given deciduous or semi-deciduous species. We plan to eventually provide photos for each species to help illustrate this, but in the meantime you will need to make note of the fullness of the canopy once the leaves or needles have grown to full size, but before they begin to fall off towards the end of the growing season. This will be what the canopy looks like at 100%. During your first year of observing, you may want to avoid estimating canopy fullness until you know what it looks like at 100% fullness.

Once you know what the canopy looks like at 100% fullness, you should estimate the changing percentage of fullness as the leaves grow larger at the beginning of the growing season (starting at 0% with bare branches before any leaves appear and increasing to 100% when the leaves become full size), and as leaves fall off and the canopy thins at the end of the growing season (starting at 100% and decreasing to 0% once all the leaves have fallen off). One trick is to estimate the percentage of sky seen through the tree canopy. If no sky is visible at 100%, then when you see half sky and half leaves within the area of the canopy, it is 50% full. A similar trick can be used for smaller plants by estimating the amount of ground you see through the canopy while looking down on a plant.

We are aware that estimates of canopy fullness by the human eye is a somewhat subjective measure, but combined with more precise measurements made from camera images, it can provide very useful information about the duration of the year over which a plant is photosynthesizing, which in turn affects the amount of CO_2 in the atmosphere.

For semi-deciduous species, the canopy fullness will not necessarily drop to 0% every year, and may not even get up to 100% every year if the environmental conditions have been extreme (for example, an extreme drought that delays leaf bud break). For these species, the extent of the canopy in a good year should be considered 100% fullness, and 0% would be the plant with all leaves dropped off.

If your plant has a dead section that never grows leaves, you can ignore that portion of the plant in estimating canopy fullness so that the plant canopy is 100% full when the live portions of the plant are fully leafed out.

7. How can I judge the percentage of full leaf size while leaves are still increasing in size?

This is a little difficult the first year you try it, but gets easier with practice. If you are in doubt, you can use a ruler to measure full size (length and/or width) of a typical leaf during summer of the first year, and then use that measure to better judge the percentage of full leaf size during the period of leaf growth in subsequent years. We are asking observers to note when leaves are less than 25%, 25-49%, 50-74%, 75-94% or 95% or more of full leaf size in order to create an estimate of the time it takes for leaves to grow to full size. Including this measure allows scientists to keep track of the length of the "green-up" period which is an important aspect of a plant's response to climate change.

8. How do I judge what percentage of the canopy is full with colored leaves or needles?

To answer this question, use the principle explained in FAQ #6, above, but consider only the colored portions of leaves and needles that are left on the plant and do not include the green portions. For instance, if the plant canopy is 100% full with leaves but about half of them are green and half are colored, you would report that 100% of the canopy is full with leaves, and 50% of the canopy is full with colored leaves. If it is windy the next day, and half of the colored leaves fall off (but none of the green leaves fall off), you would now report that 75% of the canopy is full with leaves and 25% of the canopy is full with colored leaves. As the days go on, more of the leaves change color and some fall off, and you might eventually find that only half of the leaves remain on the plant and there is no green left in them. At this point you would report that 50% of the canopy is full with leaves and 50% of the canopy is full with colored leaves. Note that the percentage of the canopy full with leaves or needles (green plus colored) should steadily decline from 100% to 0% as leaves or needles fall off. However, the percentage of the canopy full with colored leaves or needles may go up and down during this time of leaf/needle fall.

9. Why are there no intensity options provided for “Falling leaves/needles”?

There are no intensity options for this phenophase because the percentage of leaves or needles that have fallen from your deciduous plant can be calculated from the percentage of leaves or needles that remain on the plant. This is already captured in the value you reported for "What percentage of the canopy is full with leaves/needles?" for the “Leaves/needles” phenophase. See FAQ #6 above for more information.

10. How are the phenophases “Flowers or flower buds”, “Flower heads” (grasses), or “Pollen cones” (conifers) different from “Open flowers” or “Open pollen cones”?

The “Flowers or flower buds”, “Flower heads” (grasses), and “Pollen cones” (conifers) phenophases give you the opportunity to report developing flowers, flower heads, or pollen cones before you see any of the flowers or pollen cones open on your plant (flowers are not considered "open" until reproductive parts are visible). Report “Yes” for this phenophase when you see any developing flower or flower bud, fresh (unwithered) flowers, or pollen cones on your plant, ***whether they are open or closed*** (however do not include dormant flower buds enclosed in bud scales until that bud begins to break, as occurs in many northern tree and shrub species, such as forsythia). This means that whenever you report you see “Open flowers” or “Open pollen cones”, you should also be reporting that you see “Flowers or flower buds”, “Flower heads” or “Pollen cones”. For some species, flowers or pollen cones can open on sunny days and stay closed on cloudy days, in which case you should continue to report you see “Flowers or flower buds”, “Flower heads” or “Pollen cones” even when you are reporting that you do not see “Open flowers” or “Open pollen cones”. However, once all flowers or pollen cones on the plant have withered, do not report this phenophase even if the dried or withered petals or cones remain on the plant. Note that any given flowering plant species will either have single flowers that sit directly on a woody twig or at the end of a short or long stalk, or it will have many flowers on a single stalk (a flower head, flower spike, catkin or inflorescence). Report “Yes” for “Flowers or flower buds” as soon as you see something that is recognizable as a flower structure, whether it is a developing flower in the process of emerging from a twig on a tree, or a developing flower stalk emerging from the base of a wildflower.

11. How do I judge what percentage of flowers are open on a species with inflorescences?

For species where tiny individual flowers are clustered in inflorescences (flower heads, spikes or catkins), it may seem difficult to estimate the percentage of these individual flowers that are open over the entire plant. However, oftentimes the percentage of individual flowers open on a single inflorescence will be the same for all inflorescences on the plant. If this is the case, you can choose a single inflorescence, estimate the percentage of open flowers on it, and use that value to represent the entire plant. For larger plants, it is generally a good idea to check a few inflorescences (for example, one towards the bottom of the plant, one in the middle and one towards the top), and average the percentage of open flowers on each of these inflorescences to represent the entire plant.

12. How is the phenophase “Fruits” different from “Ripe fruits”?

The “Fruits” phenophase gives you the opportunity to report the presence of developing fruits before you see any of them mature or ripen on your plant. Report “Yes” for this phenophase as long as you see fruits on your plant at any stage of maturity, whether unripe and in the process of developing, or mature and ripe. This means that whenever you report you see “Ripe fruits”, you should also be reporting that you see “Fruits”. Often some fruits will ripen and be eaten or drop from the plant, while unripe fruits still remain, so you may see fruits for a long period of time with ripe fruits present on the plant during some observation days and no ripe fruits present on other observation days. Sometimes it is hard to tell when fruits first appear. Technically they are present as soon as the flower’s ovary is

fertilized, but often the ovary does not swell into something resembling a fruit for several weeks. Do not worry about missing this early stage and simply report fruits when you see the fruit as it begins to enlarge. However, once all of the fruits drop all of their seeds, do not report this phenophase even if the pods, capsules, or husks of the fruits remain (or "persist") on the plant.

13. In counting fruits, should I count all fruits on a single inflorescence as a single unit, as is done for flowers?

No, you should count individual fruits. Often not all flowers in an inflorescence will be fertilized and develop into fruit or substantial fruit abortion will occur within an inflorescence after the flowers have been fertilized. For this reason the number of fruits in an inflorescence can be highly variable. However, for some species an inflorescence will result in hundreds of tiny fruits (for example, sunflowers) and you may need to estimate the number of fruits on a plant by trying to roughly count them on a single inflorescence and multiplying by the total number of inflorescences on the plant.

Fruit structures in the plant world are very diverse and it can be difficult to identify fruits and determine ripeness. For this reason we have added a species-specific description to the fruit phenophase definitions for each species included in *Nature's Notebook* to ensure all observers are evaluating the same thing. In some cases what is described as a fruit in these definitions does not fit the botanical definition of "fruit", but you should follow the guidelines in the definition for the purposes of counting to insure consistency across all observers. Please visit the website (www.nn.usanpn.org) to find this information for each species.

14. Can I report seeing both "Unripe seed cones" and "Ripe seed cones" on the same plant at the same time?

Yes, absolutely. There are many times when you will see both unripe and ripe seed cones on the same plant at the same time. In some species, seed cones take more than a full year to mature and you could be seeing ripe seed cones from a previous year's crop at the same time you see unripe seed cones from this year's crop. Or you may even see unripe cones from the previous AND current year's crop at the same time. Just be careful not to count as "ripe" any empty cones that have already dropped all of their seeds but remain on the plant. This occurs quite frequently for some conifer species, but these empty seed cones should be ignored.

15. Why should I look for "Recent fruit or seed drop" or "Recent cone or seed drop" (conifers), and how can I tell if mature fruits or seed cones have dropped from my plant since my last visit?

Sometimes a fruit or seed cone is not ripe very long before it drops from the plant. This phenophase allows you to report that one or more fruits or seed cones ripened and dropped from the plant since your last visit. Evidence of "Recent fruit or seed drop" or "Recent cone or seed drop" may include mature fruits or seed cones on the ground below the plant that were not there on your last visit, or fruits or seed cones missing from the plant which were present on your last visit. For this phenophase, do not include the

dropping of fruits or seed cones that are clearly immature and unripe, as often happens in a heavy rain or wind storm. You should also not include fruit pods, capsules, husks, or empty seed cones that long ago dropped all of their seeds and are only now falling from the plant.

Animals

Introduction

Standardized phenophases developed by the USA-NPN for animals are outlined below. Phenophases in use for each animal group are summarized in the tables, and written definitions are included in the following sections. Each phenophase is posed to the observer as the question "Do you see/hear [phenophase]?" to which the observer records "yes", "no" or "uncertain" for each date and time of observation. More than one phenophase may be occurring in a single, individual animal at the same time. For example, if a bird is seen in the "Fruit/seed consumption" phenophase, it is also in the "Active individuals" and "Feeding" phenophases.

Intensity or abundance measures for each phenophase are included below each definition. In almost all cases this represents a count of the number of individual animals of a given species at a given site that are observed in the phenophase of interest at the time of observation. "Vocalizing" in frogs and toads is the only exception, where a series of categorical qualitative options for intensity are provided in lieu of asking observers to count the number of individuals.

A major challenge to standardization is the fact that different observers can interpret the meanings of definitions differently. We have made every effort to make the definitions as clear and complete as possible in order to minimize variation in interpretation. To this end, observers should interpret definitions literally in all situations and resist trying to second guess alternative meanings for unanticipated situations they believe the creators of the definitions might have implied but did not clearly state.

These phenophases are designed for a beginner animal observation audience, and only phenophases that are relatively easy to observe and/or evaluate are included. Phenophases for which evaluation could potentially endanger an observer (e.g., trying to catch bees in a net), or disturb an animal (e.g., following rare shorebirds closely enough to observe nest building) are not included. Additional phenophases for a more advanced audience exist in draft form and are not included in this document. Note that there are many animal groups for which phenophases have not yet been developed (e.g., bats, ticks, intertidal invertebrates, etc.). Please see the USA-NPN website (www.usanpn.org) for future updates to these protocols. Also visit the *Nature's Notebook* website (www.nn.usanpn.org) to find the specific phenophases recommended for each of over 200 animal species, including some clarifying species-specific descriptions added to phenophase definitions.

SUMMARY TABLES

Insects

Phenophase title	Mayfly	Dragonfly/Damselfly	Grasshopper	Stonefly	Tiger Beetle	Butterfly	Moth	Bee
Active adults	X	X	X	X	X	X	X	X
Adults feeding		X	X		X			
Flower visitation						X		X
Migrating adults[a]		X				X		
Mating	X	X	X	X	X	X	X	X
Egg laying		X						
Active subadults	X							
Active caterpillars[a]						X	X	
Caterpillars in tent[a]							X	
Caterpillars feeding[a]						X	X	
Dead caterpillars[a]						X	X	
Active nymphs			X					
Nymphs feeding			X					
Dead nymphs			X					
Dead adults	X	X	X	X	X	X	X	X
Individuals at a feeding station[a]						X	X	
Individuals at a light[a]					X		X	
Individuals in a net	X	X	X	X	X	X	X	

[a] excluded for species where this stage or behavior is never exhibited or is very difficult to observe

Fishes, Amphibians and Reptiles

**Phenophase title**	**Fish** (saltwater)	**Fish** (anadromous)	**Fish** (freshwater)	**Eel**	**Salamander**	**Toad/Frog**	**Alligator**	**Turtle**	**Lizard/Snake**
Individuals on land							X	X	X
Adults on land					X	X			
Individuals in water[a]							X	X	X
Adults in water[a]					X	X			
Adults in freshwater		X	X	X					
Adults in saltwater	X	X		X					
Feeding							X	X	X
Adults feeding	X	X	X	X	X	X			
Adults migrating upstream		X							
Adults migrating downstream		X	X	X					
Juveniles in saltwater	X								
Juveniles moving upstream				X					
Vocalizing						X			
Adults vocalizing							X		
Mating					X	X			
Nesting								X	
Fresh eggs					X	X			
Young individuals							X	X	X
Dead individuals							X	X	X
Dead adults					X	X			
Dead or dying adults	X	X	X	X					
Individuals on a hook	X	X	X	X					
Individuals in a net	X	X	X	X					

[a] excluded for species that are solely terrestrial

Birds and Mammals

Phenophase title	Bird (general)	Shorebird	Hummingbird	Songbird	Mammal (general)	Pinniped	Squirrel/Chipmunk	Deer/Sheep
Active individuals	X	X	X	X	X		X	X
Individuals on land						X		
Individuals in water						X		
Feeding[a]	X	X	X	X	X	X	X	X
Fruit/seed consumption[a]	X			X			X	
Insect consumption[a]	X		X	X				
Flower visitation[a]			X	X				
Nut gathering[a]	X						X	
Calls or song	X	X	X	X				
Singing males[a]			X	X				
Males vocalizing[a]								X
Male combat[a]						X		X
Mating[a]	X	X	X	X	X	X		X
Nest building[b]	X		X	X				
Young individuals					X	X	X	X
Summer coat[a]					X			
Winter coat[a]					X			
Dead individuals	X	X	X	X	X	X	X	X
Individuals at a feeding station	X		X	X				

[a] excluded for species where this stage or behavior is never exhibited or is very difficult to observe
[b] excluded for shorebirds so as not to encourage nest disturbance by observers

INSECT PHENOPHASES

Active adults

(***Mayfly /Dragonfly/Damselfly/Grasshopper/Stonefly/Tiger Beetle/Butterfly/Moth/ Bee***) One or more adults are seen moving about or at rest.

For abundance, record the number of individual animals observed in this phenophase.

Adults feeding

(***Dragonfly/Damselfly/Grasshopper/Tiger Beetle***) One or more adults are seen feeding. If possible, record the name of the species or substance being eaten or describe it in the comments field.

For abundance, record the number of individual animals observed in this phenophase.

Flower visitation

(***Butterfly/Bee***) One or more individuals are seen visiting flowers or flying from flower to flower. If possible, record the name of the plant or describe it in the comments field.

For abundance, record the number of individual animals observed in this phenophase.

Migrating adults

(***Dragonfly/Damselfly***) A swarm of adults of mostly the same species is seen flying overhead.

For abundance, record the number of individual animals observed in this phenophase.

(***Butterfly***) Multiple adults of the same species are seen flying steadily in a uniform direction without stopping.

For abundance, record the number of individual animals observed in this phenophase.

Mating

(***Mayfly/Stonefly***) A male and female are seen coupled in a mating position, usually one on top of the other.

For abundance, record the number of individual animals observed in this phenophase.

(***Dragonfly/Damselfly***) A male and female are seen coupled in a mating position, usually forming what looks like a circle with their bodies. This can be at rest or in flight.

For abundance, record the number of individual animals observed in this phenophase.

(***Grasshopper/Tiger Beetle/Bee***) A male and female are seen coupled in a mating position, usually with the male on top of the female.

For abundance, record the number of individual animals observed in this phenophase.

(***Butterfly/Moth***) A male and female are seen coupled in a mating position, usually end to end. This can occur at rest or in flight.

For abundance, record the number of individual animals observed in this phenophase.

Egg laying

(***Dragonfly/Damselfly***) A female is seen laying eggs directly onto the water surface, or attached to aquatic plants.

For abundance, record the number of individual animals observed in this phenophase.

Active subadults

(***Mayfly***) One or more subadults are seen moving about or at rest. *Species-specific description included here.*

For abundance, record the number of individual animals observed in this phenophase.

Active caterpillars

(***Butterfly/Moth***) One or more caterpillars (larvae) are seen moving about or at rest. When seen on a plant, if possible, record the name of the plant or describe it in the comments field.

For abundance, record the number of individual animals observed in this phenophase.

Caterpillars in tent

(***Moth***) Caterpillars are seen in their tent. If possible, record the name of the plant on which the tent is built or describe it in the comments field.

For abundance, record the number of individual animals observed in this phenophase.

Caterpillars feeding

(***Butterfly/Moth***) One or more caterpillars are seen feeding. If possible, record the name of the species or substance being eaten or describe it in the comments field.

For abundance, record the number of individual animals observed in this phenophase.

Dead caterpillars

(***Butterfly/Moth***) One or more dead caterpillars are seen, including those found on roads.

For abundance, record the number of individual animals observed in this phenophase.

Active nymphs

(***Grasshopper***) One or more nymphs are seen moving about or at rest. Nymphs look similar to adults, but their wings are absent or only partially developed and they cannot fly.

For abundance, record the number of individual animals observed in this phenophase.

Nymphs feeding

(***Grasshopper***) One or more nymphs are seen feeding. If possible, record the name of the species or substance being eaten or describe it in the comments field.

For abundance, record the number of individual animals observed in this phenophase.

Dead nymphs

(***Grasshopper***) One or more dead nymphs are seen, including those found on roads.

For abundance, record the number of individual animals observed in this phenophase.

Dead adults

(***Mayfly /Dragonfly/Damselfly/Grasshopper/Stonefly/Tiger Beetle/Butterfly/Moth/ Bee***) One or more dead adults are seen, including those found on roads.

For abundance, record the number of individual animals observed in this phenophase.

Individuals at a feeding station

(***Butterfly/Moth***) One or more individuals are seen visiting a feeder, feeding station, or food placed by a person.

For abundance, record the number of individual animals observed in this phenophase.

Individuals at a light

(***Tiger Beetle/Moth***) One or more individuals are seen at a light, whether flying or at rest.

For abundance, record the number of individual animals observed in this phenophase.

Individuals in a net

(***Mayfly /Dragonfly/Damselfly/Grasshopper/Stonefly/Tiger Beetle/Butterfly/Moth***) One or more individuals are seen caught in a net.

For abundance, record the number of individual animals observed in this phenophase.

FISH PHENOPHASES

Adults in freshwater

(***Anadromous/Freshwater/Eel***) One or more adults are seen in a freshwater stream, lake, or pond.

For abundance, record the number of individual animals observed in this phenophase.

Adults in saltwater

(***Saltwater/Anadromous/Eel***) One or more adults are seen in an ocean, an estuary, a saltwater or brackish wetland, or other body of saltwater.

For abundance, record the number of individual animals observed in this phenophase.

Adults feeding

(***Saltwater/Anadromous/Freshwater/Eel***) One or more adults are seen feeding. If possible, record the name of the species or substance being eaten or describe it in the comments field.

For abundance, record the number of individual animals observed in this phenophase.

Adults migrating upstream

(***Anadromous***) One or more adults are seen moving upstream, for example, in a river or fish ladder.

For abundance, record the number of individual animals observed in this phenophase.

Adults migrating downstream

(***Anadromous/Freshwater/Eel***) One or more adults are seen moving downstream.

For abundance, record the number of individual animals observed in this phenophase.

Juveniles in saltwater

(***Saltwater***) One or more juveniles are seen in a bay, an estuary, or other near-shore habitat.

For abundance, record the number of individual animals observed in this phenophase.

Juveniles moving upstream

(***Eel***) One or more immature individuals are in or entering a river mouth, moving from salt water or brackish water to freshwater stream habitat, or are moving upstream, for example, through a fish ladder, a counting station, or around a dam relatively near the mouth of a river.

For abundance, record the number of individual animals observed in this phenophase.

Dead or dying adults

(***Freshwater/Saltwater/Anadromous/Eel***) One or more dead or dying adults are seen.

For abundance, record the number of individual animals observed in this phenophase.

Individuals on a hook

(***Saltwater/Anadromous/Freshwater/Eel***) One or more individuals are seen caught on a hook.

For abundance, record the number of individual animals observed in this phenophase.

Individuals in a net

(***Saltwater/Anadromous/Freshwater/Eel***) One or more individuals are seen caught in a net.

For abundance, record the number of individual animals observed in this phenophase.

AMPHIBIAN PHENOPHASES

Adults on land

(***Salamander/Toad/Frog***) One or more adults are seen at rest or active on land.

For abundance, record the number of individual animals observed in this phenophase.

Adults in water

(***Salamander/Toad/Frog***) One or more adults are seen at rest or active in water.

For abundance, record the number of individual animals observed in this phenophase.

Adults feeding

(***Salamander/Toad/Frog***) One or more adults are seen feeding. If possible, record the name of the species or substance being eaten or describe it in the comments field.

For abundance, record the number of individual animals observed in this phenophase.

Vocalizing

(***Toad/Frog***) One or more individuals are heard vocalizing.

What is the intensity of vocalizing?

Single calls: *There is space between calls and individuals can be counted*.; **Overlapping calls:** *Calls of individuals can be distinguished but there is some overlapping of calls*.; **Full chorus:** *Calls are constant and overlapping.*

Mating

(***Salamander/Toad/Frog***) A female is seen grasped and held by a male.

For abundance, record the number of individual animals observed in this phenophase.

Fresh eggs

(***Salamander/Toad/Frog***) Eggs are seen being extruded, an egg mass is seen with jelly not expanded to full size, or embryos that are more or less spherical are seen.

For abundance, record the number of individual animals observed in this phenophase.

Dead adults

(***Salamander/Toad/Frog***) One or more dead adults are seen, including those found on roads.

For abundance, record the number of individual animals observed in this phenophase.

REPTILE PHENOPHASES

Individuals on land

(***Alligator/Turtle/Lizard/Snake***) One or more individuals are seen active or at rest on land, including individuals found under cover of a rock, log, or burrow.

For abundance, record the number of individual animals observed in this phenophase.

Individuals in water

(***Alligator/Turtle/Lizard/Snake***) One or more individuals are seen active or at rest in water, including individuals basking on a log or rock in the water.

For abundance, record the number of individual animals observed in this phenophase.

Feeding

(***Alligator/Turtle/Lizard/Snake***) One or more individuals are seen feeding. If possible, record the name of the species or substance being eaten or describe it in the comments field.

For abundance, record the number of individual animals observed in this phenophase.

Adults vocalizing

(***Alligator***) Vocal sounds produced by an adult are heard.

For abundance, record the number of individual animals observed in this phenophase.

Nesting

(***Turtle***) One or more adult females are seen nesting. This includes actual laying of eggs or excavating the nest cavity. It does not include turtles that are likely engaged only in basking.

For abundance, record the number of individual animals observed in this phenophase.

Young individuals

(***Alligator/Turtle/Egg-laying Lizard/Snake***) One or more recently hatched or young individuals are seen, living or dead, including those individuals found dead on a road.

For abundance, record the number of individual animals observed in this phenophase.

(***Lizard/Snake with live birth***) One or more recently born or young individuals are seen, living or dead, including those individuals found dead on a road.

For abundance, record the number of individual animals observed in this phenophase.

Dead individuals

(***Alligator/Turtle/Lizard/Snake***) One or more dead individuals are seen, including those found on roads.

For abundance, record the number of individual animals observed in this phenophase.

BIRD PHENOPHASES

Active individuals

(***Bird/Shorebird/Hummingbird/Songbird***) One or more individuals are seen moving about or at rest.

For abundance, record the number of individual animals observed in this phenophase.

Feeding

(***Bird/Shorebird/Hummingbird/Songbird***) One or more individuals are seen feeding. If possible, record the name of the species or substance being eaten or describe it in the comments field.

For abundance, record the number of individual animals observed in this phenophase.

Fruit/seed consumption

(***Bird/Songbird***) One or more individuals are seen eating the fleshy fruits, seeds, or cones of a plant. If possible, record the name of the plant or describe it in the comments field.

For abundance, record the number of individual animals observed in this phenophase.

Insect consumption

(***Bird/Hummingbird/Songbird***) One or more individuals are seen eating insects. If possible, record the name of the insect or describe it in the comments field.

For abundance, record the number of individual animals observed in this phenophase.

Flower visitation

(***Hummingbird/Songbird***) One or more individuals are seen visiting flowers or flying from flower to flower. If possible, record the name of the plant or describe it in the comments field.

For abundance, record the number of individual animals observed in this phenophase.

Nut gathering

(***Bird***) One or more individuals are seen taking acorns or other nuts from a plant or from on the ground. If possible, record the name of the plant or describe it in the comments field.

For abundance, record the number of individual animals observed in this phenophase.

Calls or song

(***Bird/Shorebird/Hummingbird/Songbird***) One or more individuals are heard calling or singing.

For abundance, record the number of individual animals observed in this phenophase.

Singing males

(***Hummingbird/Songbird***) One or more singing males are heard. Singing refers to stereotypical, simple or elaborate vocalizations used as part of a territorial proclamation or defense or mate attraction. It does not include relatively simple calls used for other forms of communication.

For abundance, record the number of individual animals observed in this phenophase.

Mating

(***Bird/Shorebird/Hummingbird/Songbird***) A male and female are seen coupled in a mating position, usually with the male on top of the female.

For abundance, record the number of individual animals observed in this phenophase.

Nest building

(***Bird/Hummingbird/Songbird***) One or more adults are seen constructing a nest or carrying nesting material.

For abundance, record the number of individual animals observed in this phenophase.

Dead individuals

(***Bird/Shorebird/Hummingbird/Songbird***) One or more dead individuals are seen, including those found on roads.

For abundance, record the number of individual animals observed in this phenophase.

Individuals at a feeding station

(***Bird/Hummingbird/Songbird***) One or more individuals are seen visiting a feeder, feeding station, or food placed by a person.

For abundance, record the number of individual animals observed in this phenophase.

Mammal Phenophases

Active individuals

(***Mammal/Squirrel/Chipmunk/Deer/Sheep***) One or more individuals are seen moving about or at rest.

For abundance, record the number of individual animals observed in this phenophase.

Individuals on land

(***Pinniped***) One or more individuals are seen active or at rest on land.

For abundance, record the number of individual animals observed in this phenophase.

Individuals in water

(***Pinniped***) One or more individuals are seen active or at rest in water.

For abundance, record the number of individual animals observed in this phenophase.

Feeding

(***Mammal/Pinniped/Squirrel/Chipmunk/Deer/Sheep***) One or more individuals are seen feeding. If possible, record the name of the species or substance being eaten or describe it in the comments field.

For abundance, record the number of individual animals observed in this phenophase.

Fruit/seed consumption

(***Squirrel/Chipmunk***) One or more individuals are seen eating the fleshy fruits, seeds, or cones of a plant. If possible, record the name of the plant or describe it in the comments field.

For abundance, record the number of individual animals observed in this phenophase.

Nut gathering

(***Squirrel/Chipmunk***) One or more individuals are seen taking acorns or other nuts from a plant or from on the ground. If possible, record the name of the plant or describe it in the comments field.

For abundance, record the number of individual animals observed in this phenophase.

Males vocalizing

(***Deer/Sheep***) Vocal sounds produced by an adult male are heard.

For abundance, record the number of individual animals observed in this phenophase.

Male combat

(***Pinniped***) Two or more adult males are seen wrestling or biting, or one male is seen chasing another.

For abundance, record the number of individual animals observed in this phenophase.

(***Deer/Sheep***) Two or more adult males are seen touching antlers or horns or butting heads, or one male is seen chasing another.

For abundance, record the number of individual animals observed in this phenophase.

Mating

(***Mammal/Pinniped/Deer/Sheep***) A male and female are seen coupled in a mating position.

For abundance, record the number of individual animals observed in this phenophase.

Young individuals

(***Mammal/Pinniped/Squirrel/Chipmunk/Deer/Sheep***) One or more recently born or young individuals are seen, living or dead, including those individuals found dead on a road.

For abundance, record the number of individual animals observed in this phenophase.

Summer coat

(***Mammal***) One or more individuals are seen with more than half of their coat consisting of dark hairs.

For abundance, record the number of individual animals observed in this phenophase.

Winter coat

(***Mammal***) One or more individuals are seen with more than half of their coat consisting of white hairs.

For abundance, record the number of individual animals observed in this phenophase.

Dead individuals

(***Mammal/Pinniped/Squirrel/Chipmunk/Deer/Sheep***) One or more dead individuals are seen, including those found on roads.

For abundance, record the number of individual animals observed in this phenophase.
